# “Incidence, characteristics and prognosis of cervical artery dissection-induced ischemic stroke in central Iran”

**DOI:** 10.1186/s12883-022-02754-7

**Published:** 2022-06-21

**Authors:** Mahta Ranjbar, Negin Badihian, Maryam Yazdi, Shahaboddin Milani, Marzieh Taheri, Fariborz Khorvash, Mohammad Saadatnia

**Affiliations:** 1 Department of Neurology, School of Medicine, Isfahan University of Medical Sciences, Hezar Jarib Street, Isfahan, 73461-81746 Iran; 2 Isfahan Neurosciences Research Center, Isfahan University of Medical Sciences, Isfahan, Iran; 3 Child Growth and Development Research Center, Research Institute for Primordial Prevention of Non-Communicable Disease, Isfahan University of Medical Sciences, Isfahan, Iran; 4 Interventional Cardiology Research Center, Cardiovascular Research Institute, Isfahan University of Medical Sciences, Isfahan, Iran

**Keywords:** Cervical artery, Dissection, Stroke, Incidence, Prognosis

## Abstract

**Objectives:**

Ischemic stroke is the most common presentation of cervical artery dissection (CAD). Information regarding CAD-induced stroke is scarce, especially in the Middle East. Here we investigated the incidence of CAD-induced stroke, its characteristics, and the clinical course in central Iran.

**Methods:**

This is an observational study conducted in the city of Isfahan, Iran. We recruited patients with ischemic stroke during 2017–2019. We analyzed characteristics of the CAD-induced stroke patients with regards to the involved vessel (internal carotid artery dissection (ICAD) or vertebral artery dissection (VAD)). We assessed functional outcome (modified Rankin Scale [mRS]) and recanalization status after 1 year of follow-up.

**Results:**

Among 3630 ischemic stroke patients, 51(1.4%) had CAD-induced stroke (mean age: 41.8 ± 12.6; 40.4% female; 28 and 19 ICAD and VAD cases, respectively). The crude incidence rate of CAD-induced stroke was 1.20/100,000/year (0.66/100,000/year and 0.45/100,000/year for strokes due to ICAD and VAD, respectively). mRS ≤ 2 was present in 63.8% of the patients after 1 year of follow-up. History of exercise during the last days before stroke occurrence was associated with a better follow-up mRS (β = -3.1, p-value: 0.037). Administration of anticoagulant or double-antiplatelets was related neither to mRS nor recanalization results. Trauma (27.7%), smoking (21.3%), and headache disorders/migraine (21.3%) were the most common reported factors.

**Conclusion:**

We found a crude incidence rate of 1.20/100,000/year for CAD-induced stroke. Trauma, smoking, and headache disorders were the most common reported factors among our patients. CAD-induced stroke had a favorable long-term prognosis regardless of the type of the involved vessel or the used medication.

## Introduction

Stroke is one of the main causes of mortality and disability worldwide [1]. Ischemic strokes account for 87% of all strokes in the United States and 76–88% of strokes in Iran [2–7]. During the last decades, the incidence of ischemic strokes among young adults has been increased [8].

Ischemic strokes may occur due to different causes, including atherosclerosis of large arteries, occlusion of small vessels, cardioembolic events, arterial dissection, hyper coagulopathy, vasculitis, hemodynamic instabilities, etc. [9]. The accurate diagnosis of stroke etiology is challenging but is of great importance to determine the treatment approach and prognosis and establish prevention strategies [10]. The prevalence of stroke etiologies varies significantly between young and old individuals. Cervicocerebral artery dissection is one of the rare causes of ischemic stroke in the older population but a major cause among young adults [11].

Dissection is defined as the separation of arterial wall structural elements that will further form an intramural hematoma in the affected area [12]. It would cause narrowing of the arterial lumen, decreased blood flow, increased chance of embolism formation, and consequently non-ischemic local symptoms [12, 13]. Cervicocerebral artery dissection can occur as an extracranial (cervical) or intracranial condition [12]. Cervical artery dissection (CAD) is responsible for almost 10–25% of strokes in patients under 45 years [10, 12]. CAD may arise in internal carotid artery and with lower frequency in vertebral artery [14]. It may present with a wide range of symptoms including headache, neck pain, tinnitus, ataxia, nausea, Horner’s syndrome, ischemic symptoms (e.g. stroke and transient ischemic attack (TIA)), and death [15, 16].

It has been suggested that almost 67–77% of CAD patients experience ischemic stroke or TIA [10]. Due to the increased implementation of advanced and sensitive imaging and diagnostic tools, the diagnosis of CAD has been increased in recent years [10]. However, only limited studies have evaluated the incidence of CAD-induced stroke. Moreover, most of the previous studies have been conducted in Western countries and there is a lack of studies evaluating this condition in the Middle East. No studies have evaluated the incidence of CAD-induced stroke and its characteristics in Iran hitherto. Also, the management, treatment, and long-term outcome of this condition are areas of debate yet [10, 17]. Hence, new epidemiological studies on the incidence, clinical characteristics and outcomes of CAD-induced stroke are needed [12]. In the present study we aimed to investigate the incidence of CAD-induced stroke, its prevalence, characteristics, and the clinical course among Iranian patients for the first time.

## Materials and method

### Study participants

This is an observational study conducted from March 2017 to March 2019 in the city of Isfahan, Iran. Isfahan is the third most populated city of Iran, with a population of over 2 million, and is in the central region of Iran. In brief, we identified all patients > 18 years with a documented diagnosis of ischemic stroke admitted to the university and non-university hospitals in the city of Isfahan during the study period. Stroke was defined as an infarction in the central nervous system caused by ischemia based on clinical, pathological, or neuroimaging evidence suggesting a permanent injury [18]. The diagnosis of acute ischemic stroke was approved by 2 board certified neurologists at each center after a thorough evaluation of symptoms and neuroimaging findings. We further grouped patients into different subtypes of ischemic stroke. Ischemic stroke subtypes were defined according to the phenotyping system of ASCOD (A for atherosclerosis; S for small-vessel disease; C for cardiac pathology; O for other cause, and D for dissection) [11].

We then retrieved patients with the diagnosis of CAD-induced ischemic stroke (D1, potentially causal based on ASCOD grades for dissection) for further investigations [11]. CAD patients were diagnosed based on the symptoms and neuroimaging findings of the presence of an aneurysmal dilatation (mural hematoma, long tapering stenosis, double lumen, an intimal flap or occlusion) ≥ 2 cm above the carotid bifurcation, or a long tapering stenosis in the internal carotid or vertebral artery after recanalization [17]. Written informed consents were obtained from all CAD-induced stroke cases and they were excluded from further analyses in case of lack of consent or unavailability of information. We followed up CAD-induced stroke cases for 1 year (until March 2020). The study was approved by the ethics committee of Isfahan University of Medical Sciences (ethics code: IR.MUI.MED.REC.1397.309).

### Study design

This study was consisted of two major steps. First, we recorded information regarding age and ischemic stroke subtype for all ischemic stroke patients admitted to the Isfahan hospitals during March 2017 to March 2019. We used this information to tabulate the prevalence of ischemic stroke in patients < 45 years and ≥ 45 years, as stroke in patients < 45 years of age is defined as stroke in young population [19], based on the ASCOD classification. We also used this information to calculate incidence rate of CAD-induced stroke.

In the second step, we approached patients with a confirmed diagnosis of CAD-induced stroke to evaluate the characteristics and incidence of this type of stroke based on the involved vessel. We also asked patients to return to the clinic after 12 months of stroke occurrence to assess functional outcomes of CAD-induced stroke and evaluate the recanalization status. Hence, we recorded information regarding stroke presenting symptoms, the main involved artery and its side, neuroimaging findings, degree of baseline disability based on modified Rankin Scale (mRS) [20], symptoms progression, complete blood count (CBC), electrocardiogram (ECG), vasculitis and homocysteine tests results, and medication history for CAD-induced stroke patients. We also recorded history of cough or intercourse just before stroke happening, history of recent infection including common cold symptoms and upper respiratory tract infection, exercise, or delivery during the last days before stroke occurrence, and history of head trauma during the last month before stroke occurrence, for dissection patients. History of migraine, smoking, drug and alcohol abuse, and oral contraceptive pill (OCP) use were also recorded.

In the follow up session (after 12 months of stroke occurrence), we assessed the follow up-mRS and recanalization status. Unfavorable functional outcome was defined as mRS > 2. Recanalization was defined as ≥ 50% relative improvement in the detected stenosis following the treatment.

### Statistical analysis

Continuous variables were expressed as means ± standard deviation (SD) and categorical variables as frequencies (%). Categorical variables were compared using chi-square test or Fisher’s exact test, as appropriate. We used Mann–Whitney U test to compare continuous variables. To identify associated factors with mRS at the follow-up, ordinal logistic regression analyses were performed with adjustment for baseline mRS. Crude incidence rates were calculated and then adjusted for age and gender by the direct method to the WHO reference population [21]. Confidence intervals for Poisson distribution were calculated. We performed statistical analyses using Stata version 14 (STATA: released 14, STATA corporation 2015 College Station, TX, USA). An alpha cut off threshold of < 0.05 was considered statistically significant.

## Results

During the first 2 years of study period, 3630 patients were diagnosed with ischemic stroke in Isfahan. Based on ASCOD classification, “other causes” were the most common causes of stroke among patients, accounting for 44.1% of all cases (Table 1). Among all patients, 4.5% of cases were < 45 years.

**Table 1:** Prevalence of ischemic stroke sub-types based on ASCOD classification in the 2 age groups

Variable	< 45 years	≥ 45 years	Total	*P*-value^†^
**Stroke sub-types**				
Atherosclerosis	16(4.8)	314(95.1)	330(100)	< 0.001
Small-vessel disease	15(1.7)	868(98.3)	883(100)	
Cardiac pathology	30(3.9)	734(96.0)	764(100)	
Other causes	72(4.5)	1530(95.5)	1602(100)	
Dissection	32(62.7)	19(37.2)	51(100)	

Percentages are presented as between rows

^†^: resulted from Chi-square test

Of 3630 patients with the diagnosis of ischemic stroke, 51 (1.4%) patients were CAD-induced stroke cases, including 32 patients < 45 years old. Overall, CAD was responsible for 19.4% of all ischemic stroke cases in patients < 45 years (Table1). The crude incidence rate of CAD-induced stroke was 1.20/100,000/year (95% CI: 0.91–1.57) for the 2 years of study period. Age-standardized incidence rate (ASIR) by WHO reference population was 1.19/100,000/year (95% CI: 0.93–1.43) for CAD-induced stroke.

Among 51 CAD patients, complete information was available for 47 patients. The crude incidence rate of stroke due to internal carotid artery dissection (ICAD) and vertebral artery dissection (VAD) were 0.66 /100,000/year (95% CI: 0.45–0.95) and 0.45/100,000/year (95% CI: 0.28–0.70), respectively. ASIR was 0.65/100,000/year (95% CI: 0.46–0.84) for ICAD-induced stroke and 0.44/100,000/year (95% CI: 0.28–0.60) for VAD-induced stroke. Only 1 out of 47 patients reported a previous history of stroke and none of the patients reported history of TIA.

ICAD was diagnosed in 28 (59.5%) patients and VAD was present in 19 (40.4%) patients. Patients’ baseline characteristics are presented in Table 2. The mean age of patients diagnosed with ICAD and VAT were 42.8 ± 14 and 40.3 ± 10.3 years, respectively (*p*-value = 0.416). Right side artery involvement was reported in 13 (46.4%) and 26 (55.3%) patients from the ICAD and VAD groups, respectively (p-value = 0.137).

**Table 2:** Patients’ baseline characteristics

	**ICAD**	**VAD**	**Total**	***P***-value^**†**^
**Age (**mean ± SD)	42.8 ± 14	40.3 ± 10.3	41.8 ± 12.6	0.416
**Gender [**female]	13(46.4)	6(31.6)	19(40.4)	0.309
**Baseline mRS (**mean ± SD)	2.3 ± 1.3	2.7 ± 1.2	2.5 ± 1.2	0.623
**mRS at 1 year-follow up (**mean ± SD)	2.0 ± 1.2	1.8 ± 1.3	1.9 ± 1.2	0.673
**Affected side [**Right]	13(46.4)	26(55.3)	26(55.3)	0.137
**Headache at presentation**	17(60.7)	16(84.2)	33(70.2)	0.084
**Neck pain at presentation**	10(35.7)	10(52.6)	20(42.6)	0.250
**Hemiparesis at presentation**	4(21.0)	18(64.2)	22 (46.8)	0.101
**Progression of symptoms**	3(10.7)	6(31.6)	9(19.1)	0.129
**Addiction [heroin, methadone, and opium]**	4(14.3)	5(26.3)	9(19.1)	0.453
**Type of the addiction**				
Heroine	1(3.6)	0(0.0)	1(2.1)	0.643
Methadone	0(0.0)	1(5.3)	1(2.1)	
Opium	2(7.1)	4(21.1)	6(12.8)	
**Alcohol**	2(7.1)	1(5.3)	3(6.4)	1.000
**Smoking**	5(17.9)	5(26.3)	10(21.3)	0.496
**OCP use**	1(7.7)	0(0.0)	1(5.3)	1.000
**Infection**	3(10.7)	1(5.3)	4(8.5)	0.638
**Intercourse**	0(0.0)	0(0.0)	0(0.0)	-
**Exercise**	2(7.1)	1(5.3)	3(6.4)	1.000
**Cough**	3(10.7)	1(5.3)	4(8.5)	0.638
**Delivery**	0(0.0)	0(0.0)	0(0.0)	-
**Previous Headache/ Migraine**	6(21.4)	4(21.1)	10(21.3)	1.000
**Hypertension**	4(14.2)	2(10.5)	6(12.7)	0.705
**Diabetes**	1(3.5)	2(10.5)	3(6.3)	0.338
**Trauma**	8(28.6)	5(26.3)	13(27.7)	0.865
**Type of the trauma**				
Minor	3(37.5)	1(20.0)	4(30.8)	1.000
Major	5(62.5)	4(80.0)	9(69.2)	
**Vasculitis result**				
Negative	11(84.6)	9(100.0)	20(90.9)	0.494
Positive	2(15.4)	0(0)	2(9.1)	
**Homocysteine result**				
Negative	10(76.9)	9(100.0)	19(86.4)	0.240
Positive	3(23.1)	0(0.0)	3(13.6)	
**Medication**				
Double anti platelet	21(75.0)	16(84.2)	37(78.7)	0.718
Anti-coagulant	7(25.0)	3(15.8)	10(21.3)	
**New headaches after dissection**	8(28.6)	8(42.1)	16(34.0)	0.337
**Change in the pattern of headaches**	3(50.0)	2(50.0)	5(50.0)	1.000
**Vascular findings**				
Significant stenosis	3(17.6)	2(16.7)	5(17.2)	0.808
Mobile clot	2(11.8)	0(0.0)	2(6.9)	
Total occlusion	12(70.6)	10(83.3)	22(75.9)	
**Imaging findings**				
Cerebellar infarct	0(0.0)	8(42.1)	8(17.0)	< 0.001
Brain stem	0(0.0)	9(47.4)	9(19.1)	
Hemispheric	25(89.3)	1(5.3)	26(55.3)	
Bilateral	2(7.1)	1(5.3)	3(6.4)	
No infarct	1(3.6)	0(0.0)	1(2.1)	

*ICAD* Internal Carotid Artery Dissection, *VAD* Vertebral Artery Dissection, *mRS* Modified Rankin scale, *OCP* Oral Contraceptive Pill. Percentages are listed in parentheses

^†^: resulted from Chi-square test (or Fisher’s exact test when expected frequencies were less than 5) for categorical variables and Mann–Whitney’s test for continues variables

Hemispheric infarct was the most common reported imaging finding seen in 26 (55.3%) CAD induced-stroke cases. CAD risk factors are presented in Table 2. History of previous headache was reported by 10 (21.3%) patients and five out of these 10 patients reported a change in the pattern of their headaches after stroke occurrence.

Headache was the most common reported symptom at the time of hospital admission, seen in 33 (70.2%) cases. We found no statistically significant difference between the frequency of headache as the presenting symptom between ICAD and VAD cases (60.7% and 84.2%, respectively; *p*-value = 0.084).

At the time of hospital admission, the mean mRS was 2.3 ± 1.3 and 2.7 ± 1.2 among patients with ICAD and VAD, respectively (*p*-value = 0.623). Mean mRS at the time of follow up was 1.96 ± 1.28 among CAD-induced stroke patients, regardless of type of the dissection. An unfavorable follow-up mRS was observed in 11 (39.2%) and 6 (31.5%) patients suffering ICAD and VAD, respectively (*p*-value = 0.209).

Hemispheric infarct, brainstem infarct, and cerebellar infarct were the most common reported findings observed in 55.3%, 19.1% and 17% of all patients, respectively. Patients with ICAD and VAD had different neuroimaging findings, summarized in Table 2.

Baseline mRS was a significant predictor of follow-up mRS (Spearman’s *r* = 0.68, *p*-value < 0.001). Therefore, to investigate predictive value of other variables for follow-up mRS we used ordinal logistic regression model adjusted for baseline mRS.

Follow-up mRS was significantly lower in patients who reported exercising during the last days before stroke occurrence (*p*-value = 0.037). We found no other factors associated with lower follow-up mRS (Table 3). None of the patients experienced stroke recurrence, symptomatic dissection, or TIA during the one year follow up.

**Table 3:** The relationships between possible risk factors and prognostic factors and mRS at 1 year-follow up

	**Baseline mRS**	**mRS at 1 year-follow up**	**Percent change (%)**	**Patients with mRS > 2 / Total patients**	**β** ^a^ **(SE)**	***P*-value**
**Sex**						
Female (ref.)	2.2 ± 1.3	1. ± 1.2	9.6 ± 34.2	8/19	-0.1(0.6)	0.824
Male	2.7 ± 1.2	2.0 ± 1.3	26.3 ± 35.7	9/28		
**Age**						
< 45 (ref.)	2.8 ± 1.2	2.1 ± 1.2	24.3 ± 32.2	12/31	1.8(0.4)	< 0.001
≥ 45	2.0 ± 1.2	1.7 ± 1.3	10.4 ± 41.2	5/16		
**Vessel**						
Carotid (ref.)	2.4 ± 1.3	2.0 ± 1.2	10.7 ± 31.5	11/28	0.5(0.6)	0.341
Vertebral	2.7 ± 1.2	1.9 ± 1.3	32.7 ± 38.3	6/19		
**Side**						
Right (ref.)	2.6 ± 1.3	2.0 ± 1.4	21.3 ± 43.4	11/26	0.04(0.5)	0.948
Left	2.4 ± 1.2	1.9 ± 1.1	17.4 ± 23.8	6/21		
**Headache**						
No (ref.)	2.8 ± 1.1	2.6 ± 1.0	2.3 ± 35.1	8/14	-1.2(0.6)	0.058
Yes	2.4 ± 1.3	1.6 ± 1.2	26.9 ± 33.9	9/33		
**Neck pain**						
No (ref.)	2.6 ± 1.4	2.2 ± 1.4	11.6 ± 35.7	13/27	-0.7(0.6)	0.193
Yes	2.3 ± 1.1	1.6 ± 1.0	30.4 ± 33.7	4/20		
**Progression of symptoms**						
No (ref.)	2.5 ± 1.3	1.8 ± 1.3	22.0 ± 38.4	13/38	1.0(0.7)	0.136
Yes	2.5 ± 1.1	2.3 ± 1.2	9.2 ± 18.8	4/9		
**Addiction**						
No (ref.)	2.5 ± 1.3	1.9 ± 1.2	20.7 ± 36.5	14/38	0.6(0.7)	0.391
Yes	2.3 ± 1.3	2.0 ± 1.5	14.8 ± 33.8	3/9		
**Alcohol**						
No (ref.)	2.5 ± 1.3	1.9 ± 1.2	18.6 ± 34.6	16/44	-0.1(1.2)	0.964
Yes	2.3 ± 0.5	1.6 ± 1.5	33.3 ± 57.7	1/3		
**Smoking**						
No (ref.)	2.6 ± 1.3	2.0 ± 1.2	17.2 ± 34.2	15/37	-0.3(0.7)	0.695
Yes	2.1 ± 1.1	1.5 ± 1.3	28.3 ± 41.6	2/10		
**OCP use**						
No (ref.)	2.2 ± 1.4	1.8 ± 1.2	10.2 ± 35.2	7/18	N/A	N/A
Yes	3.0 ± 0.0	3.0 ± 0.0	0.0 ± 0.0	1/1		
**Infection**						
No (ref.)	2.6 ± 1.3	2.0 ± 1.3	20.2 ± 36.7	17/43	0.5(0.9)	0.604
Yes	1.5 ± 0.5	1.2 ± 0.5	12.5 ± 25.0	0/4		
**Exercise**						
No (ref.)	2.5 ± 1.3	2.0 ± 1.2	16.9 ± 33.6	16/44	-3.1(1.5)	0.037^*^
Yes	3.0 ± 1.0	1.3 ± 1.5	58.3 ± 52.0	1/3		
**Cough**						
No (ref.)	2.6 ± 1.3	2.0 ± 1.3	20.2 ± 36.7	16/44	0.5(0.9)	0.604
Yes	1.5 ± 0.5	1.2 ± 0.5	12.5 ± 25.0	1/3		
**History of trauma**						
No (ref.)	2.4 ± 1.2	1.9 ± 1.2	16.9 ± 27.5	12/34	-1.0(0.7)	0.167
Yes	2.8 ± 1.4	1.9 ± 1.3	26.6 ± 52.3	5/13		
**Trauma type**						
Minor (ref.)	2.2 ± 1.7	2.2 ± 1.7	-12.5 ± 62.9	2/4	N/A	N/A
Major	3.1 ± 1.2	1.7 ± 1.3	44.0 ± 38.9	3/9		
**Vasculitis test result**						
Negative (ref.)	2.8 ± 1.2	2.2 ± 1.3	22.0 ± 29.7	9/20	-0.9(1.6)	0.574
Positive	2.5 ± 0.7	1.5 ± 0.7	33.3 ± 47.1	0/2		
**Homocysteine test result**						
Negative (ref.)	2.8 ± 1.2	2.1 ± 1.3	25.4 ± 31.7	7/19	0.9(1.1)	0.425
Positive	3.0 ± 1.0	2.6 ± 0.5	8.3 ± 14.4	2/3		
**Previous headache/ Migraine**						
No (ref.)	2.6 ± 1.2	2.0 ± 1.2	18.6 ± 36.3	15/37	-0.2(0.7)	0.781
Yes	2.3 ± 1.4	1.7 ± 1.5	23.3 ± 35.3	2/10		
**New headache**						
No (ref.)	2.4 ± 1.2	2.0 ± 1.3	15.3 ± 36.7	13/31	-0.5(0.6)	0.383
Yes	2.7 ± 1.3	1.8 ± 1.3	27.9 ± 35.7	4/16		
**Change in the pattern of headaches**						
No (ref.)	1.6 ± 1.1	1.0 ± 1.0	26.6 ± 43.4	0/5	N/A	N/A
Yes	3.0 ± 1.4	2.4 ± 1.6	20.0 ± 29.8	2/5		
**Recanalization**						
No (ref.)	2.2 ± 1.2	1.9 ± 1.5	13.6 ± 48.7	4/11	0.02(0.7)	0.978
Yes	2.5 ± 1.4	2.0 ± 1.3	19.0 ± 29.5	10/25		
**Medication**						
Double anti platelet (ref.)	2.5 ± 1.2	1.9 ± 1.2	21.0 ± 38.2	14/37	0.3(0.7)	0.617
Anti-coagulant	2.5 ± 1.6	2.0 ± 1.5	14.1 ± 25.4	3/10		

*mRS* Modified Rankin scale, *OCP* Oral Contraceptive Pill, *SE* Standard Error, *N/A* Not Applicable as there was not enough data to fit ordinal logistic model

^a^ Coefficient regression resulted from ordinal logistic regression model included bassline mRS as covariate

Regarding management, anti-coagulant and double antiplatelet medications were prescribed for 10 (21.3%) and 37 (78.7%) patients, respectively, based on the physicians’ discretion. It should be noted that double antiplatelet therapy was done for the first 3 months after stroke occurrence, and it was switch to single antiplatelet therapy after that. Among 47 CAD-induced stroke patients, 35 agreed to be assessed for the recanalization status after 1 year of dissection occurrence. Of these, recanalization occurred in 20 out of 28 patients treated with double antiplatelet and five out of 7 patients treated with anti-coagulants. We found no statistically significant relationship between type of the prescribed medication and the recanalization status (OR = 1.00, 95% CI: 0.16,6.26, *p*-value = 1.000).

## Discussion

In this study, we found a crude incidence rate of 1.20/100,000/year for CAD-induced ischemic stroke in the city of Isfahan, Iran. CAD was the etiology of stroke among 19.4% of patients < 45 years and 0.5% of patients ≥ 45 years. ICAD and VAD were responsible for 59.57% and 40.43% of the CAD-induced strokes, respectively. The only factor showed to be associated with a favorable follow-up mRS was history of exercise during the last days before stroke occurrence. None of the double antiplatelet or anti-coagulant medications were superior to the other one in improving follow up-mRS nor they increased the chance of later recanalization.

Different studies have reported an annual incidence rate of 2.6–5 per 100,000 individuals for CAD among young patients; the incidence would gradually decrease in older ages [10, 12, 22–24]. Previous population-based studies have reported the incidence rate of 1–1.5 and 2–3 per 100,000 individuals for VAD and ICAD, respectively [10, 22–24]. Information on the incidence rate of CAD presenting with stroke is scarce and most of the previous studies have investigated the incidence of CAD only. The best study that we can compare our results with is the study by Bejot et al. [17]. They reported a crude incidence rate of 2.97/100,000/year for CAD-induced cerebrovascular events (stroke and TIA). Their results showed a corresponding incidence rate of 1.21/ 100,000/year for cerebrovascular events due to ICAD, and 1.87/100,000/year for cerebrovascular events due to VAD [17]. We found lower incidence rates in our study. We speculate that these differences are mainly due to the methodological differences, although race and ethnicity could also be contributing factors. In the study by Bejot et.al, both TIA and stroke cases were recorded; however, we only looked at ischemic stroke cases. Also, their study population was considerably lower compared to us (total number of 1368 patients with cerebrovascular events, among them 27 patients with CAD-induced stroke).

The prevalence of new or recurrent ischemic stroke among CAD patients is unclear. In some studies, up to 50–60% of cases that were further diagnosed by CAD were initially presented with ischemic stroke [10, 22, 25, 26]. CAD-induced stroke is categorized as “stroke of other determined cause” in TOAST (Trial of Org 10,172 in Acute Stroke Treatment) classification. CAD is suggested to be the most common cause of stroke in this category [27]. The incidence of stroke of other determined cause was reported to be 5.11/100,000/year in a study conducted in Iran in 2017. However, the exact proportion of dissection patients was not reported in that study or other studies conducted in the Middle East [28]. In a study conducted in Saudi Arabia, among 85 stroke patients aged 18–45 years, 17.6% were classified as stroke of other determined cause with only 1 patient with VAD [29]. In another study in Lebanon, the prevalence of stroke of other determined cause category was reported to be 10% [30].

Headache, hemiparesis, and neck pain were the most common presenting symptoms of CAD-induced stroke among our patients. Similarly, headache and neck pain were among the most common presenting symptoms in patients with CAD in the previous studies [17, 22, 31]. Trauma (27.7%), smoking (21.3%) and headache disorders/migraine (21.3%) were the most common reported factors among patients in our study. These results are in the same vein with reports from the previous studies. In the study by Bejot et al. smoking and migraine were reported by 37% and 29.6% of the CAD-induced stroke/TIA, respectively [17]. Arauz et al. reported smoking as the most common risk factor (36%) among 130 CAD patients [32]. In another study conducted among 459 CAD patients, smoking, trauma and migraine were reported by 29%, 26.7% and 25.8% of the participants, respectively, and were consequently the most common reported risk factors [26].

Here we found a favorable follow-up mRS among 63.82% of CAD-induced stroke cases. We did not observe a statistically significant difference regarding mean follow up-mRS between patients with ICAD and VAD-induced-strokes. Also, VAD did not show to be associated with an improved follow-up mRS compared to ICAD. Favorable short- and long-term outcomes (mRS ≤ 2) among CAD patients have been reported by most of the previous studies. Young age of the CAD cases (mean age around 35 to 45) and fewer concurrent underlying medical conditions have been suggested as possible explanations for more favorable outcome in this group [17]. In the study by Arauz et al., 27% of ICAD patients and 78% of VAD patients reported a favorable outcome by 6 months after dissection occurrence. They also found that a favorable outcome was more frequent among VAD patients compared to those with ICAD [32]. In another study by Lee et al., a favorable clinical outcome was reported in 92% of 48 CAD patients with a mean follow-up of 7.8 years. In the study by Bejot et al., among 19 patients with stroke related to spontaneous CAD, 84.2% had a favorable mRS at three-months after stroke occurrence [17]. The slightly lower percentage of patients showing a favorable outcome in our study could be due to the study target population (only CAD patients presenting with ischemic stroke), different follow-up periods, and a positive history of recent trauma in some cases.

Follow-up mRS was significantly lower in patients who reported exercising during the last days before stroke occurrence in our study. We assume that patients who reported exercising during the last days before stroke occurrence, were probably engaged in sports activity more regularly compared to others. Similar to what we observed, in a study lack of physical activity prior to stroke was found as an independent predictor of poor outcome in a year from stroke [33].

Regarding management in our study, double-antiplatelets and anticoagulants were prescribed for 78.7% and 21.3% of the patients, respectively. Recanalization occurred in 71.4% of the patients after 1 year follow-up. Previous studies reported an equivalent efficacy for antiplatelet and anticoagulant treatment in CAD-induced stroke patients, but no study have compared the efficacy of short-term dual-antiplatelets and anticoagulants after acute ischemic strokes [10, 34]. Generally, the decision on the prescribed medication depends on various patient factors, history, and list of medications [35].

In the current study, the most common neuroimaging findings among CAD-induced stroke patients were hemispheric infarct, brainstem infarct, and cerebellar infarct, with different frequency of these findings among ICAD and VAD individuals. Most of the ICAD patients presented with hemispheric infarct, while brainstem infarct and cerebellar infarct were the most common findings among VAD patients. In the study by Lee et al., of 60% of the CAD patients who showed an infarct in their neuroimaging, cerebral infarct was documented in 83% of the VAD patients and 47% of the ICAD patients [22]. Cerebral infarct has been reported as the most common neuroimaging finding in other previous studies as well [36–38].

To the best of our knowledge, this is the first study that assesses the incidence of CAD-induced stroke in Iran and presents patients factors in this group. The main limitation of our study is that we were not able to assess the recanalization status in all CAD-induced stroke patients as some of them were not willing to undergo further neuroimaging studies. Also, we were not able to collect the exact duration of hospitalization and cigarette pack/year in each patient. It should be noted that our findings on the incidence rate of CAD-induced stroke might not be generalizable to the whole Iranian population and further studies from other regions of Iran are needed.

## Conclusion

The crude incidence rate for CAD-induced ischemic stroke was 1.20/100,000/year in our study. CAD-induced stroke was higher among patients under 45 years. Headache, hemiparesis, and neck pain were the most common presenting symptoms in CAD-induced stroke patients. History of exercise during the last days before stroke occurrence was associated with a better functional outcome after 1 year. History of trauma, smoking, and previous headache/migraine were the most common reported factors. Hemispheric infarct was the most common neuroimaging finding in stroke patients with ICAD, while cerebellar and brain stem infarcts were more common findings among those with VAD. Most of the patients reported a mRS ≤ 2 after 1 year of follow-up indicating the good prognosis of ischemic stroke due to CAD. None of the double-antiplatelet or anticoagulant medications were superior to the other one with regards to follow-up mRS. In most of the patients, recanalization was occurred by 1 year of follow-up, regardless of the medical management. This study provides valuable findings on the characteristics of CAD-induced ischemic stroke in the Iranian population. More population-based studies with longer follow-ups are recommended to better understand the specific characteristics and prognostic factors of CAD-induced stroke**.**

## Data Availability

The data that support the findings of this study are available from the corresponding author upon request from qualifying researchers. The data are not publicly available due to privacy and ethical restrictions.
